# The implementation of the nursing process in lower‐income countries: An integrative review

**DOI:** 10.1002/nop2.410

**Published:** 2019-11-01

**Authors:** Mojgan Lotfi, Vahid Zamanzadeh, Leila Valizadeh, Mohammad Khajehgoodari, Mehdi Ebrahimpour Rezaei, Mohammad Amin Khalilzad

**Affiliations:** ^1^ Department of Medical Surgical Nursing Faculty of Nursing and Midwifery Tabriz University of Medical Sciences Tabriz Iran; ^2^ Department of Pediatric Nursing Faculty of Nursing and Midwifery Tabriz University of Medical Sciences Tabriz Iran; ^3^ Master of Science Library and Information Science Department of Information Technology Faculty of Nursing and Midwifery Tabriz University of Medical Sciences Tabriz Iran; ^4^ Student of Laboratory Sciences Tabriz Azad University of Medical Sciences Tabriz Iran

**Keywords:** integrative review, nursing, nursing process

## Abstract

**Aims:**

This review study aimed to investigate the strategies for implementing the nursing process in the clinical practice and the assessment of the implementation rate of this process in clinical settings of lower‐income countries.

**Design:**

An integrative review.

**Method:**

The search was conducted of EMBASE, MEDLINE, CINAHL, Scopus and ISI databases from 1975–July 2018. Following the formation of the research team, two researchers independently selected the eligible studies; finally, 39 articles were approved by the research team for this study.

**Results:**

The researchers identified three themes: Effects of implementing the NP in clinical settings, Development and application of electronic software in the NP and Factors affecting the implementation of the NP. This review revealed that nurses and nursing managers in hospitals are interested in implementing the nursing process in the form of widely and continuously. But the necessary infrastructure, such as manpower, electronically or manually tools, has not yet been provided, and the implementation of the nursing process is done either imperfectly or not done.

**What does this paper contribute to the wider global clinical community?**

The nursing process is not implemented nursing in Iran, Ethiopia, Kenya, Taiwan and generally in lower‐income countries, for the following three reasons:

- Inadequate knowledge of nursing faculty members from the nursing process.
- Lack of necessary infrastructures in hospitals.
- Lack of support from nursing institutions (Nursing Organization and The Nursing Board).

## 1 INTRODUCTION

One of the most important care standards is the nursing process (NP) that helps nurses making a clinical decision. The nursing process is a scientific method that uses scientific reasoning, problem‐solving and critical thinking for delivering holistic and quality nursing care (Wagoro & Rakuom, 2015). Implementation of the nursing process in the clinical settings improves the quality of nursing care, enhances the level of nurses “knowledge, improves the quality and quantity of nurses” documentation and increases their job satisfaction and self‐efficacy (Hagos, Alemseged, Balcha, Berhe, & Aregay, 2014; Potter & Perry, 2017; Semachew, 2018). In this process, the nurse needs to assess and identify the problem, review the existing solutions, select and implement the best option and ultimately evaluate them (Potter & Perry, 2017). All the steps are done with the patient's participation (Potter & Perry, 2017). The NP is a tool for helping the nurse to make appropriate clinical decision‐making and critical thinking (Ghanbari, Monfared, Hoseinzadeh, Moaddab, & Sedighi, 2017). The NP as the most effective nursing care planning and implementation method leads to effective communication between the nurses and the patients, increasing both the participation of the patient in self‐care and the quality of nursing care (Muller‐Staub, Needham, Odenbreit, Lavin, & van Achterberg, 2008). Furthermore, it is functional and adaptable in any clinical setting and manages the time of care, and it also prevents the occurrence or repetition of the mistakes (Paese, Sasso, & Colla, 2018). The NP is used to provide services to all clients, including individuals, families, groups or communities, and it is unique to nursing (Potter & Perry, 2017). The main element of the NP is the nursing diagnosis that helps the nurses in guiding nursing care and in promoting the documentation process (Paans, Nieweg, van‐der‐Schans, & Sermeus, 2011). Despite the importance of this process for patients and nurses, the conditions for its implementation in low‐income countries (IPNA & Residents/nationals of these countries qualify for low‐income country rates, 2018) are unclear and dissatisfaction with nursing care has been widely reported in these countries (Ghafouri Fard, Haririan, Aghajanloo, Akbari, & Shirvani, 2012; Lotfi, Zamanzadeh, Valizadeh, & Khajehgoodari, 2019; Rajabpoor et al., 2018; Semachew, 2018).

## 2 BACKGROUND

Nursing as a process was described for the first time by Lydia Hall in 1955. She introduced 3 STEPs for nursing as a process: Observation, Administration of care and Validation (de la Cuesta, 1983), and Orlando, in 1961, used the term "NP" in his theory and described the process as patient behaviour, nurse response and nursing practices (Orlando, 1961). In 1967, Yura and Walsh described the NP in four steps: Assessment, Planning, Implementing and Evaluating in their book. Authors, such as Iyer, Taptich, & Bernocchi‐Losey (1995), Phaneuf, López, & Ruíz (1993) or Alvarez (1987), considered the nursing diagnosis, which was traditionally seen as part of the nursing assessment as a separate phase and described the process as containing five phases (Potter & Perry, 2017). Nowadays, the NP is a systematic problem‐solving with five steps: assessment, diagnosis, planning, implementation and evaluation to identify, prevent and treat actual or potential health problems and promote wellness (Potter & Perry, 2017). For the emphasis of the American Nursing Association (ANA) on passing the course of nursing process after formal education to upgrade their qualifications, approval of a law on the validation of nursing care based on patient care plans by the Joint Commission on the Accreditation of Hospitals (JCAH) in the 1970s and increased nurses' concern for their development as a profession due to the developments in nursing education leading to the nursing process was seen as an important means for that development (Yura & Walsh, 1978).

In a decade, it was used as a teaching tool in educational settings, and when this process was used in hospitals, it appeared useful and effective for most countries (de la Cuesta, 1983).

The acceptance and emergence of the nursing process in England were related to the professional dimension. In this period, dissatisfaction wave from the nursing care provided in the United Kingdom. Major factors of dissatisfaction include denial of a task‐orientated approach to nursing, the lack of individualized care, the low level of nurses’ job satisfaction and the superficial nature of the nurse–patient communication. The nursing process, as an antidote for the treatment of toxins, destroyed most nursing dissatisfactions in the country (de la Cuesta, 1983; Walton, 1986).

In lower‐income countries (IPNA, & Residents/Nationals of These Countries Qualify for Low‐Income Country Rates, 2018) due to the dissatisfaction of care provided by nurses, poor quality of care and nurses' dissatisfaction from their profession have led use the nursing process; since 1980, studies have begun on the implementation of the NP in the clinical settings (de la Cuesta, 1983; Vanaki & Zamanzadeh, 1994; Zamanzadeh, Valizadeh, Jabbarzadeh‐Tabrizi, Behshid, & Lotfi, 2015). In several studies, the usefulness of this process has been confirmed in accordance with the context of different countries (Semachew, 2018; Zamanzadeh et al., 2015).

However, the acceptance and application of the nursing process are clear in the high‐income countries (Di Mauro, Vanalli, Alberio, & Ausili, 2018), but in low‐income countries after about 40 years of the development of and usefulness of this process in these countries, it is not yet known how much the nursing process is implemented, the acceptability of the nursing process and the importance of this process in the clinic settings (Fernández‐Sola et al., 2011; Wagoro & Rakuom, 2015; Zamanzadeh et al., 2015).

## 3 AIM

To evaluate the strategies for implementing the NP in the clinical practice and the assessment of the implementation rate of this process in clinical settings of lower‐income countries.

Research question:

Is the nursing process implemented with specific strategies in lower‐income countries in the form of widely and continuously?

## 4 METHODS

### 4.1 Design

The integrative review (Whittemore & Knafl, 2005) was applied in this study. Considering that scientific information is increasing in all fields and professions nowadays, practitioners do not have enough time to review all the information on their interested background to get the most valuable information; the integrative review (IR) method is an approach that by combining different methods and examining all the findings of particular issues or subjects, and provides useful and valuable information to the researcher or practitioners on that subject (Whittemore & Knafl, 2005). Mixed studies review (MSR) can be more appropriate for decision‐makers and practitioners by providing a rich and practical understanding of complex health interventions and programmes (Pace et al., 2012). In this research, we used the Whittemore and Knafl's (2005) integrative review framework stages, which included Problem identification, Literature search, Data evaluation, Data analysis and Presentation.

### 4.2 Stage 1: Problem Identification

The first step in the review method is the clear identification of the problem; then, variables of interest are defined theoretically and practically. The NP has five consecutive steps, and nurses can improve the quality of their care by implementing this process. The NP as a systematic and dynamic way to deliver nursing care included five interrelated steps: assessment, diagnosis, planning, implementation and evaluation. This process is a cycle that never ends, the guideline that ensures good nursing care and improves patient outcomes (Fernández‐Sola et al., 2011; Potter & Perry, 2017). There are many effective factors in the implementation of the nursing process including knowledge of faculty members, nursing managers and nurses about the NP, the interest and attitude of nurses towards the implementation of the process, manager's support in the implementation of the NP and implementing the NP in clinical settings (Potter & Perry, 2017; Shoorideh & Ashktorab, 2011; Zamanzadeh et al., 2015).

### 4.3 Stage 2: Literature Search

The research question was designed based on the SPICE (setting, perspective, intervention, comparison and evaluation) framework that is more valuable than PICO (population, intervention, comparison and outcomes) framework with two statistically significant changes (Cooke, Smith, & Booth, 2012). These changes included dividing the population component into both “setting” and “perspective” and “evaluation” instead of outcomes (Andrew, 2006; Crumley & Koufogiannakis, 2002). These new concepts of the SPICE framework authenticate that data practice is a social science, not a hard science, and incorporates other concepts such as “outputs” and “impact” together with less tangible effects of a library or instructional intervention (Andrew, 2006). SPICE framework is a more appropriate framework for health and social sciences (Andrew, 2006; Eldredge, 2001) and helps practitioners to identify their practice‐based questions (Andrew, 2006). This framework was also used for matching the research design to the question, inclusion and exclusion criteria and guide the database search strategy (Andrew, 2006).

All databases were searched using the terms: (Nursing Process OR Nursing Process software OR Nursing Diagnosis OR Nursing assessment) AND (quality of care OR implementation strategies OR outcome) AND (nurse OR nurses OR registered nurses) AND (clinical setting OR hospital unit).

To determine the type of extracted studies, the method of searching, and determining the inclusion and exclusion criteria, the research group was formed on 5 May 2018. In the first, two researchers (MK and ME (health librarian)) independently searched for articles in EMBASE, MEDLINE, CINAHL, Scopus and ISI databases from 1975–July 2018 without any language restrictions. Grey literature searching was conducted using professional databases and dissertations (Masters and Ph.D.). Then, the final papers were extracted according to the inclusion and exclusion criteria of the study (Table 1). The reasons for choosing low‐income countries including Iran, Brazil, Bolivia, Taiwan, Ethiopia, Mexico and Egypt in this study were that the challenges of nursing care in their clinical settings were similar (Fernández‐Sola et al., 2011; Ledesma‐Delgado & Mendes, 2009; Rajabpoor et al., 2018; Semachew, 2018; Yeh et al., 2009).

**Table 1:** Inclusion and exclusion criteria

Inclusion criteria	Studies that: Published between 1975–2018 Conducted the implementation NP (manually or electronically) in the clinical setting Evaluated the relationship between the NP or nursing diagnosis and outcomes Assessment of the nursing record systems Published on the documentation of the NP Published on evaluated nursing diagnosis, facilitators and barriers to the implementation of the NP Published related to clinical settings in lower‐income countries including Iran, Brazil, Bolivia, Taiwan, Ethiopia, Mexico and Egypt Published in English or Persian language
Exclusion criteria	Studies that: NP is evaluated outside the hospital Presented as a lecture Containing conference proceedings or letters to the editor.

**Figure 1:**
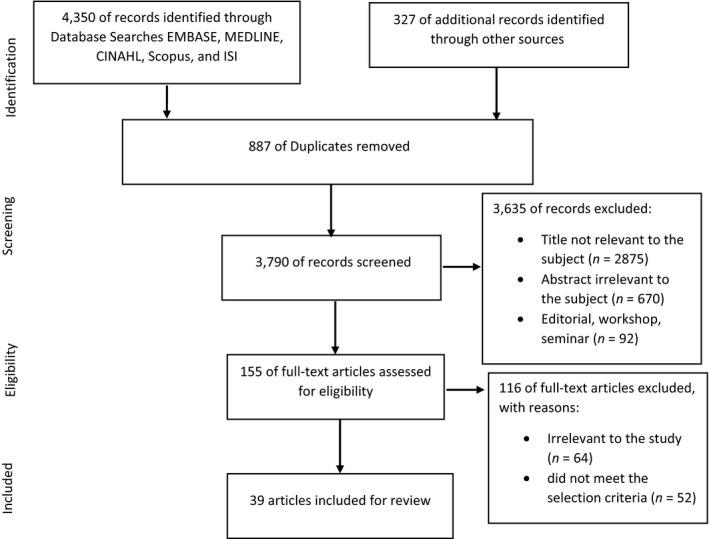
PRISMA flow chart showing article selection stages

The initial search resulted in 4,350 records from databases and 327 records from grey literature and reference by reference based on the search terms. Subsequently, 887 papers were duplicates and excluded from the study and the total records identified were 3,790. Finally, 39 articles were identified consistent with the inclusion and exclusion criteria. The process of identifying, evaluating and selecting articles is presented based on preferred reporting items for systematic reviews and meta‐analyses (PRISMA) (Moher, Liberati, Tetzlaff, & Altman, 2009) in Figure 1.

### 4.4 Stage 3: Data Evaluation

Critical appraisal of methodological features is complex to evaluate the quality of studies in integrative reviews (Whittemore & Knafl, 2005). Due to lack of guidelines for evaluating research quality in integrative reviews, the Mixed Methods Appraisal Tool (MMAT) was adopted as it helps overcome challenges associated with evaluating the methodological quality of varied studies (Hong et al., 2018; Pace et al., 2012; Whittemore & Knafl, 2005). We used this method to evaluate the quality of selected studies and also to increase the validity of the study.

The Mixed Methods Appraisal Tool (MMAT) seems to be a useful and unique tool for evaluating MSR (qualitative, quantitative and mixed methods) with scores varying between 25% (meeting one criterion)–100% (meeting all four criteria) (Crowe & Sheppard, 2011). The MMAT is designed for the appraisal stage of systematic mixed studies reviews and permits to appraise the methodological quality of five categories to studies of qualitative research, randomized controlled trials, non‐randomized studies, quantitative descriptive studies and mixed methods studies (Crowe & Sheppard, 2011; Hong et al., 2018). This tool is recommended by the National Institute of Excellence in Health Services in Quebec (INESS) and increasingly popular because of their potential for addressing complex interventions and phenomena, specifically for assessing and improving clinical practice (Hong, Gonzalez‐Reyes, & Pluye, 2018).

The eligibility of articles was discussed in the research team. To appraise the papers selected in this study, at first, two reviewers independently evaluated the quality of the papers with the MMAT method, and then, it was discussed in the research team, and in cases where there were disagreements, the articles and scores of the two reviewers were examined to until consensus was reached. Articles with a score of less than 50 percent were excluded from the review. None of the studies was excluded during the quality appraisal of the articles by the research team.

According to the evaluation of studies based on MMAT, it was found that the quality of studies was moderate. The quality scores for the studies are included in Tables 2, 3 and 4. Data collection was performed in most quantitative studies using researcher‐made questionnaires; therefore, the necessity of designing and psychometrics of a functional tool seems essential for evaluating the implementation of the NP.

**Table 2:** Effects of implementing the nursing process in clinical settings

Author(s)/ year & country	Research aim	Study design	Study population	Data collection	Setting	Results/key findings	MMAT score
(Rasouli, Hagiamiri, Mahmoudi, & Mostoufian, 1996) Iran	In order to use the nursing process to prevent and reduce pressure ulcers in orthopaedic wards	Semi‐experimental	Nurses (*n* = 30) Convenience sampling method	Multichoice questionnaire	Orthopaedic wards of hospitals affiliated to Tehran University of Medical Sciences	Training based on the nursing process increased nurses' knowledge and change in the degree of pressure ulcer.	50%
(Rahgooy, Vanaki, Golestan, & Soulati, 1999) Iran	For the effect of nursing process education on the quality of care provided to psychiatric patients	Experimental with control group	Nurses (*n* = 44) Random sampling method	The QualPaks standard checklist	Razi Psychiatric Center of Tehran	The quality of nursing care after the training of the case group was improved from moderate to good rather than before training and the control group.	50%
(Vanaki & Zamanzadeh, 1994) Iran	In order to investigate the effect of nursing process implementation using problem‐based recording in quantity and quality of nurses documentation	Semi‐experimental	Nurses (*n* = 42) Convenience sampling method	Researcher checklist	The Imam Hussein Hospital Tehran	The urgent need for an accurate documentation system and the improvement in the quality and quantity of nursing documentation after the implementation.	75%
(Akbari & Farmahani, 2011) Iran	In order to influence the nursing process on the quality of care of schizophrenic patients	Semi‐experimental with control group	Nurses (*n* = 30) Convenience sampling method	The QualPaks standard checklist	Shaheed Lavasani Hospital of Tehran	The quality of care provided in the physical–psychological needs and the relationship of the patient well‐promoted.	50%
(Oshvandi, PourYousef, & Bikmoradi, 2013) Iran	To effect of clinical education by exploration on the skill of using nursing process by nursing students	Semi‐experimental with control group	Nursing students (*n* = 38) Convenience sampling method	Checklist	Surgical wards of Besat hospital in Hamadan	Exploration education has a more positive effect on nursing students' clinical skills learning than conventional education.	50%
(Aein & Frouzandeh, 2012) Iran	Determining the effectiveness of conceptual mapping in nursing process learning in clinical nursing education in children	Semi‐experimental	Nursing students (*n* = 30) Convenience sampling method	Checklist	Shahrekord Children's Hospital	Quantitative and qualitative evaluation of conceptual maps showed a statistically significant progression of students in understanding the nursing process, which improved from the weak level on the first day to the excellent level on a ninth day.	50%
(Habibzadeh, Khajehali, Khalkhali, & Mohammadpour, 2013) Iran	To analyse the impact of evidence‐based nursing education on five scopes of nursing process among nursing students.	Quasi‐experimental study with control group	Nursing students (*n* = 48) Convenience sampling method	Checklist	Orthopaedic wards of Orumieh Hospital	Members of the intervention group had better performance in all five scopes of the nursing process in comparison with the control group.	50%
(Rastian, Borzabady Farahani, Rasouli, Sarbakhsh, & Niromand, 2014) Iran	To determine the effect of nursing process implementation on quality of nursing care of patients hospitalized in surgical wards	Quasi‐experimental study with before–after design	Nurses (*n* = 48) Purposive sampling method	Quality patient care scale (QualPacS) checklist	Surgical wards of teaching hospitals affiliated to Yasoj Medical University	Nursing process implementation can improve the quality of nursing care of patients in surgical wards.	50%
(Zamanzadeh et al., 2015) Iran	Carried out to assess the key challenges associated with the implementation of the nursing process	Systematic review	125 articles were selected from databases of Iranmedix, SID, MagIran, PUBMED, Google scholar and ProQuest, which were assessed using the main keywords of nursing process and nursing process systematic review	‐	Tabriz University of Medical Sciences	Main challenges include intangible understanding of the concept of the nursing process, different views of the process, lack of knowledge and awareness among nurses related to the execution of process, supports of managing systems and problems related to recording the nursing process.	50%
(Khosravan, Saadat, & Moradi Kosha, 2014) Iran	To determine the effect of nursing process on job satisfaction of employed nurses	Semi‐empirical study	Nurses (*n* = 47) by census methods	Manokian questionnaire and process registration checklists of Hasson and Arnetz	Intensive care units of Gonabad hospitals	The nursing process is beneficial in clinical settings also leads to job satisfaction of nurses.	75%
(Lotfi Mojgan, 1998) Iran	In order to effect the implementation of the nursing process on the quality of nursing care from patients admitted in critical care units	Quasi‐experimental study	Patients (*n* = 52) Convenience sampling method	Questionnaire made by researcher	Intensive care units	The implementation of the nursing process showed a small effect on the quality of nursing care in critical care units.	75%
(Rahmani et al., 2016) Iran	To determine the effect of nursing process the way "accessible care cards" on patients' satisfaction from care in intensive care units	Cross‐sectional interventional study	patients (*n* = 76)	Questionnaire made by researcher	Intensive care units in the Golestan hospital in Ahwaz	Implementation of the nursing process, in a manner of available cards, led to an increase in patient satisfaction compared with the routine manner	50%
(Yeh et al., 2009) Taiwan	To develop and implement NPSSC enabling computerized documentation for nursing home residents, evaluate the efficiency of NPSSC, obstacles to the use of the NPSSC and assess nurse users’ satisfaction with the NPSSC	Quasi‐experimental one group pre‐/post‐test	Nurses (*n* = 27) & medical records (*n* = 396)	Satisfaction Questionnaire and checklist efficacy of the NPSSC	Nursing home residents in Taiwan	Obstacles use of the NPSSC was identified. The use of the NPSSC significantly improved nursing documentation in that resident's records were organized and consistent and nurses were able to complete a comprehensive care plan in 48 hr. Nurses' satisfaction with nursing documentation increased.	100%
(Semachew, 2018) Ethiopia	To evaluate the implementation of the nursing process at three randomly selected governmental hospitals	Hospital‐based descriptive and retrospective study design	Records (*n* = 338) Samples were proportionally allocated for each hospital based on the total number of inpatients in the last 6 months	Nursing process implementation checklist	Governmental hospitals	Nursing process documentation should be promoted and nursing managers should supervise the implementation of the nursing process and facilities its implementation.	50%
(Fernández‐Sola et al., 2011) Bolivia	To identify those factors that favours or hinder the NP implementation and the SCP both in the clinical areas and academic environment	Participatory action research	Research team and nurses (*n* = 35)	Meetings with key informants, Interviews, observation and workshops	Hospitals and universities	The implementation of standard care plans requires much effort. Making the most of cooperation projects to make improvements and undertake scientific research is an excellent opportunity to promote the nursing profession in less‐developed countries.	75%
(Lopes et al.., 2010) Brazil	To evaluate how the nursing process has been registered at a Brazilian teaching hospital	Descriptive and retrospective study	Medical records (*n* = 68).	Researcher‐made form	Women's Hospital CAISM/UNICAMP, a public tertiary university hospital	All steps in the nursing process were not documented, especially the nursing diagnosis.	50%
(Lima & Kurcgant, 2007) Brazil	To understand the meanings nurses at a university hospital attribute to the implementation process of the Nursing Diagnosis Classification System (DEn) as a phase in the Nursing Care System	Qualitative methodological approach	Nurses (*n* = 8)	Interviews	Hospital	Throughout the process, with the gradual increase in theoretical–practical training and participation, the collaborators became agents of change, disclosing a positive transformation in their feelings, after their initial discomfort and unfavourable perception about the implementation of the nursing diagnosis in the NCS.	50%
(Ledesma‐Delgado & Mendes, 2009) Mexican	To understand the meanings attributed to the nursing process by clinical nurses at a Mexican hospital	Qualitative study based on grounded theory	Nurses (*n* = 16)	Semi‐structured interviews, participant observation and document research	Medicine unit at the General Hospital	The nursing process was described as a nursing care routine that different from what was taught at the university.	75%

**Table 3:** Development and application of electronic software in "the nursing process"

Author(s)/ year & country	Research aim	Study design	Study population	Data collection	Setting	Results/key findings	MMAT score
(Paese et al., 2018) Brazil	To structure and organize the data and information of the computerized nursing process through ICNP® version 2.0 for emergency units	Hybrid research with quantitative and technological production in 5 stages	Nurses	The data organized in Excel worksheets and divided by human systems and degrees of complexity	Emergency care units	In the emergency ward, it was found that ICNP has a strong and solid form for the development of the computerized nursing process able to support nurses in safe decision‐making to improve the quality of health care.	50%
(Dal G.T Sasso, Peres, & Silveira, 2006) Brazil	Describing the development of the computerized nursing process in intensive therapy from the CIPE version Beta 2 and demonstrating the contributions to the improvement of nurse care	Methodological study and technological production in three phases	Nurses (*n* = 5)	Semi‐structured questionnaire	CCU ward of hospital	The programme allows a continuous learning and explicit clinical decision‐making of the nurse.	75%
(G. T. M. Dal Dal Sasso et al., 2013) Brazil	To examine the relationship between the data and information in the nursing process, which were computerized according to ICNP version 1.0 and to establish associations between detailed clinical evaluations of each human system and diagnoses, interventions and patient outcomes.	Technological product and a methodological study in three main steps	Nurses and students in the ICU and Emergency units of hospital	General meetings to review associations and enter data.	ICU and Emergency units of hospital	The success of this technology lies in its achievement of the integration of research, professional practice and teaching. Also, provide support for decision‐making with associations between clinical evaluations, diagnoses, interventions and the results.	75%
(Sperandio & Evora, 2009) Brazil	To demonstrate that high‐tech solution can give nurses more time for direct patient care	Descriptive/exploratory study	Nurses (*n* = 11)	‐	School hospital of the state of Sao Paulo	He results showed that the incorporation of the mobile, wireless computer technology in the nursing care process provided an environment with mobility for actions and made communication and documentation of the care easier.	50%
(Silva, Evora, & Cintra, 2015) Brazil	To develop software to support decision‐making in the selection of nursing diagnoses and interventions for children and adolescents.	Methodological applied study based on software engineering, as proposed by Pressman, developed in three cycles	Nursing professionals and students	‐	University hospital in Paraiba	This software improves the quality of nursing care and nursing decision‐making skills, and it also facilitates the documentation of nurses.	50%
(Mazlom & Rajabpoor, 2014) Iran	To design and assess the local nursing process computerized software	Two phases of software design and assessment:	Students and nurses (*n* = 20) were convenience sampling method	Researcher‐made questionnaire	ICU ward of Ghaem hospital in Mashhad	Application of this software leads to increased accuracy, decreased error and shared labour that is counted as factors promoting patient care services.	75%
(Sayadi & Rokhafroz, 2013) Iran	To study nursing students’ opinions about a nursing process mobile software (as a means for facilitating nursing process implementation) for bedside use	Pre‐experimental study	Nursing students (*n* = 30)	Researcher‐made questionnaire	Cardiology ward of Golestan hospital in Ahvaz	Using this software and can improve the clinical skills of nursing students and encourage them to learn and implement the nursing process.	50%
(Lima et al., 2018) Brazil	To construct a mobile technology capable of assisting the nurse in performing nursing prescription in neonate patients	Methodological study with a qualitative approach in three phases	Nurses	Ruby on Rails, IONIC 2, Postgres SQL and Amazon EC2	Neonatal units of hospital	Computerized tool the nursing process, facilitated the data collection, diagnostic reasoning and identification and grouping of the clinical signs indicated by the newborn in neonatal units.	75%
(Domingos et al., 2017) Brazil	To identify in the literature the evidence on the use of nursing process applied to software	Integrative review	Articles (*n* = 23)	The selected articles were evaluated for the level of evidence	Federal University of Viçosa	Two categories of analysis include development and use of software and, in general, the use of NP software enhances nursing practice.	50%

**Table 4:** Factors affecting the implementation of the nursing process

Author(s)/year & country	Research aim	Study design	Study population	Data collection	Setting	Results/key findings	MMAT score
(Akbari & Shamsi, 2011) Iran	To diagnose the nursing process barriers from the perspective of the intensive care units’ nurses	Cross‐sectional descriptive study	Nurses (*n* = 63) based on cluster‐random sampling method	Questionnaire	Hospitals of Tehran	The most important individual and managerial barriers were determined to include lack of sufficient information about the concept of the nursing process and lack of belief in doing the patient care according to the nursing process and lack of enough time for doing the nursing process due to the excessive number of the patients.	50%
(Nohi, Karimi, & Najmaei, 2010) Iran	Determining the different barriers to practical application of nursing process from the point of view of nursing managers and nursing students	Descriptive study	All nurse managers (*n* = 103) and senior nursing students (*n* = 50) with Census sampling	Researcher‐made questionnaire	Hospitals of Kerman University of Medical Sciences	Most of the obstacles in both groups were related to the barriers of execution of 75%, and the minimum barriers to scientific barriers were 12 per cent.	50%
(AtashzadehShoorideh & Ashktorab, 2011) Iran	To explore the factors that may influence the implementation of nursing process by nurses	Qualitative research based on grounded theory	Nurses, nurse educators and nurse managers (*n* = 36) with purposeful and theoretical sampling	Semi‐structured interviews	Shahid Beheshti University of Medical Sciences	Factors which influence the implementation of the nursing process by nurses are varied and complex and related to personal and managerial factors.	75%
(Ghafouri Fard et al., 2012) Iran	Identifying barriers to implementing nursing process from the point of view of nursing faculty members and students	Cross‐sectional descriptive study	Faculty members (*n* = 14) and students (*n* = 48)	Researcher‐made questionnaire	Nursing and Midwifery Faculty of Zanjan	The lack of adequate knowledge of the concept of the nursing process, its inadequate learning and the lack of supervision and follow‐up of nursing officials in implementing this process were identified as the most important barriers to individual and managerial management in the implementation of the nursing process.	50%
(Mohammadi et al., 2016) Iran	To determine the nursing process barriers from the view of the nurses and nurse managers	Descriptive cross‐sectional study	Nurses (*n* = 73) and nurse managers (*n* = 17) with Census sampling	Researcher‐made questionnaire	Surgical wards of Imam Reza hospital	It was determined that not having knowledge of the concept of the nursing process as the most important individual barrier and not having enough time to implement the nursing process was identified as the most important management barrier due to a large number of patients.	50%
(Matbouei, Mohammadi, & Zargarzadeh, 2013) Iran	To assess nurses and nurse manager's point of view about the barriers to documenting the nursing diagnosis	Descriptive cross‐sectional approach to problem‐solving	Nurses and nursing managers (*n* = 97)	Researcher‐made questionnaire	Trbiat‐e‐Modares university of Tehran	The main obstacles to recording nursing diagnosis are numerous written works by nurses, transfer of non‐nursing activities to nurses, not assigning privileges to nurses who identify and record nursing diagnosis, failure to perform a care system for each patient individually and lack of in‐service training.	50%
(Rajabpoor et al., 2018) Iran	To determine the barriers to the implementation of the nursing process from the viewpoint of the faculty members, nursing managers, nurses and nursing students	Analytical cross‐sectional study	Nursing lecturers and students (*n* = 90) and nurses and nursing managers (*n* = 134) were selected by convenience sampling method	Research‐oriented questionnaire	Mashhad University of Medical Sciences	The lack of a checklist for recording the process in the medical records of the patients, the high number of patients under the care of each nurse and the lack of a principal training of the nursing process during their studentship were the most important obstacles to the implementation of the nursing process.	75%
(Lee, 2005) Taiwan	To explore factors that may affect nurses’ use of nursing diagnoses in charting standardized nursing care plans in their daily practice.	Qualitative research	Nurses (*n* = 12)	Interviews	Medical Center in Taiwan	Nurses were reluctant to match the patient's condition with nursing diagnosis and care required due to lack of knowledge about nursing diagnosis, nursing care programme and interventions.	75%
(Takahashi et al., 2008) Brazil	To identify the difficult and easy aspects of performing the different stages of the nursing process, according to the reports of nurses	Descriptive/exploratory study	Nurses (*n* = 83)	Questionnaires (both structured and open‐ended)	The university hospital belonging to that institution	The most important obstacle to the implementation of the nursing process is the lack of knowledge associated with theoretical and practical knowledge phases of the nursing process. Nurses also had difficulty in applying and recording nursing diagnosis and evaluation.	75%
(Shewangizaw & Mersha, 2015) Ethiopia	To assess factors affecting implementation of nursing process among nurses	Cross‐sectional study	Nurses (*n* = 105) randomly sampling method	Self‐administered pre‐tested semi‐structured questionnaire and observational checklist	Arbaminch General Hospital	The study has identified a lack of facility from organizational factors, economic status of the patient to collect material for nursing care, early discharge, lack of cooperation and complicated problems from patient‐related factors and level of knowledge were among those factors highly affecting nursing process implementation.	75%
(Manal & Bayoumy, 2014) Egypt	To explore barriers and facilitators for execution of nursing process from nurses' perspective.	Descriptive exploratory design	Nurses (*n* = 148) Convenience sampling method	Questionnaire	Najran General Hospital and King Khalid Hospital	Data collection identified by the majority as the difficult phase. Nurses identified barriers related to work as the most commonly encountered barriers.	50%
(Aseratie, Murugan, & Molla, 2014) Ethiopia	To assess factors affecting implementation of nursing process among nurses in selected governmental hospitals	Cross‐sectional quantitative study	Nurses (*n* = 202) Simple random sampling method	Questionnaire	Governmental hospitals at Addis Ababa	Organizational factors, patient‐related factors and level of knowledge and skill were among those factors highly influenced nursing process implementation.	50%

### 4.5 Data analysis

The data analysis stage is one of the most difficult aspects and potentially fraught with error. Similar data are categorized and grouped, after compared data. Then, these coded categories are compared which improves the analysis and synthesis process (Whittemore & Knafl, 2005). The first, to manage data for a better understanding and enhance the visualization of patterns, shows the relationships between primary data sources, and the following characteristics are considered as the initial subgroups: author, country, year, study design, data collection and results. Then, data synthesis from the selected studies was coded by highlighting relevant parts of the text and assigning code words to these areas. Following this, an iterative process was used to develop categories by combining codes. Descriptive themes were attached to each category and are discussed in data comparison (Dal Sasso et al., 2013; Frigstad, Nøst, & André, 2015; Ghafouri Fard et al., 2012; Ledesma‐Delgado & Mendes, 2009; Mazlom & Rajabpoor, 2014; Mohammadi, Ghafori Fard, & Esmaeilivand, 2016; Rahmani, Alijani, Dashtbozorge, & Haghighizadeh, 2016; Rajabpoor et al., 2018; Semachew, 2018; Zamanzadeh et al., 2015).

#### 4.5.1 Ethic

Given that this review article was part of a Ph.D. thesis, a code of ethics was obtained from the Ethics Committee with number: IR.TBZMED.REC.1397.170.

## 5 RESULTS

Of the 39 studies identified in this review, 18 (46%) focused on the effects of implementing the nursing process in the clinical setting, nine focused (23%) addressed the development and application of software to support the NP, and 12 (31%) discussed factors that affected the implementation of the NP. The papers studied were mainly quantitative papers, and fourteen articles were conducted using descriptive, exploratory and cross‐sectional design. Twelve articles were conducted using the quasi‐experimental or RCT research design, five articles were conducted using technological products and methodological studies, five articles were conducted using qualitative research design, and three articles were conducted using the review articles. Most quantitative studies used researcher‐made questionnaires to collect the required information.

After data are compared with each other to identify the specific patterns of studies and the precise and important themes in them, three themes were identified that the description of each is given below:

1. Effects of implementing the NP in clinical settings
2. Development and application of electronic software in the NP
3. Factors affecting the implementation of the NP.

## 6 EFFECTS OF IMPLEMENTING THE NP IN CLINICAL SETTINGS

Of the 39 studies reviewed, 18 studies were studied in this scope (Table 2). The implementation of the NP in the clinical practice improves the quality of nursing care, increases the quantity and quality of the curriculum, improves nurses “knowledge, improves the quality and quantity of nurses” documentation and increases job satisfaction and self‐efficacy.

## 7 DEVELOPMENT AND APPLICATION OF ELECTRONIC SOFTWARE IN THE NP

Nine studies were examined on designing NP software and its application (Table 3). The NP electronically documentation is a reliable tool for measuring the quality of diagnostic documents, interventions and nursing outcomes and more efficient than a manual documentation system. The development of this tool and its application can help in decision‐making and quality of care. It also reduces errors and increases the care of nurses in inpatient care.

## 8 FACTORS AFFECTING THE IMPLEMENTATION OF THE NP

Out of 12 studies on the factors influencing the implementation of the NP, 6 papers examined the barriers to the implementation of the NP, 6 studied on the factors affecting the implementation of the process, and its barriers have been discussed (Table 4). In general, the factors affecting the implementation of the NP can be divided into two categories, individual factors and management factors.

The following factors were considered as individual factors: inadequate knowledge of faculty members from the concept of NP and consequently poor learning by students and nurses and nurses' lack of desire to implement NP in the clinical practice due to low knowledge.

Management factors include shortage or lack of infrastructure for the implementation of NP, incomplete documentation system in nursing reports, nurses' high workload, nursing staff shortages in hospitals, weak in in‐service training, lack of supervision of managers on implementation of NP, lack of punishment and encouragement for the implementation of the process and lack of support nursing institutions for the implementation of the NP.

### 8.1 Stage 4: Presentation

In the final stage of the framework, more precise details of the primary sources and evidence as a logical chain to provide a result consistent with the findings provide for the reader of the review (Whittemore & Knafl, 2005).

Studies that conducted the NP manually or electronically in the clinical practice did not indicate the way of patient's assessment, signs and symptoms specific to any nursing diagnosis, the number of nursing diagnoses that were recorded in the software or that they were trained and the number of diagnoses that nurses identified after completing the process at the wards (Cho & Park, 2003; Dal Sasso et al., 2013; Di Mauro et al., 2018; Dykes et al., 2007; Frigstad et al., 2015; Lima & Kurcgant, 2007; Lima, Vieira, & Nunes, 2018; Lopes, Higa, Reis, Oliveira, & Christóforo, 2010; Mazlom & Rajabpoor, 2014; Minthorn & Lunney, 2012; Paese et al., 2018; Rahmani et al., 2016; Saba & Feeg, 2005; Sayadi & Rokhafroz, 2013; Semachew, 2018; Silva, Évora, & Cintra, 2015; Zamanzadeh et al., 2015).

Studies of Mazlom and Rajabpoor (2014) and Sayadi and Rokhafroz (2013) was done on the software design of the NP, and the software testing was limited to one ward of a hospital and was not used in other wards or other hospitals in Iran. And their software became for the lack of implementation of this software in hospitals, the lack of necessary infrastructures and the lack of supporting by responsible institutions. Virtually, the NP in Iran is not executed either manually or electronically. Moreover, except for the study of Dal Sasso et al. (2013) and Saba and Feeg (2005), information on the use of others designed the NP software in other countries is not available.

## 9 DISCUSSION

Based on the findings of this study, it was found that the nursing process can be used continuously and extensively in lower‐income countries. The findings of the study indicate that the nursing process is consistent with the context of clinical settings in these countries (Manal & Bayoumy, 2014; Semachew, 2018; Zamanzadeh et al., 2015).

The NP is accepted as a care standard with the stages of assessment, nursing diagnosis, planning, implementation and evaluation in the world. The NP has been of great help for nurses in the development of the nursing profession, nursing research and facilitating management activities in nursing (Potter & Perry, 2017).

The implementation of this process increases patients' satisfaction due to an increase in patient–nurse communication, improves the quality of nursing care and documentation. If the nursing process is implemented electronically, it saves time and nursing errors are reduced (Ghafouri Fard et al., 2012; Rajabpoor et al., 2018; Semachew, 2018; Takahashi, Barros, Michel, & Souza, 2008). But, in lower‐income countries for three reasons, the NP is not conducted extensively and continuously at the hospitals or performed very poorly: low proficiency of faculty members from the concept of the NP, lack of necessary infrastructure and lack of supporting nursing institutions and managers (Rahmani et al., 2016; Rajabpoor et al., 2018; Semachew, 2018).

Faculty members do not provide the necessary conditions for transferring student learning from the knowledge stage to the application stage and higher levels of Bloom's taxonomy (Anderson & Sosniak, 1994; Johnsen, Fossum, Vivekananda‐Schmidt, Fruhling, & Slettebo, 2016).

The lack of infrastructures such as lack of appropriate nursing documentation system manually, lack of software NP, poor HIS in hospitals, lack of standard tools for assessing nursing care based on NP and lack of tools supervising the implementation of the NP is the second reason that the nursing process is not implemented in these countries (Rahmani et al., 2016; Rajabpoor et al., 2018; Semachew, 2018).

The third reason for not institutionalizing the NP in lower‐income countries (lack of supporting nursing institutions and managers) includes lack of belief in improving the quality of nursing care based on the NP, lack of supervision on the implementation of the NP at the time of implementation as a pilot, lack of appropriate encouragement and punishment system, increased workload of nurses by caring for a large number of patients, shortage of nursing staff and lack of awareness of the concept of the NP (Akbari & Farmahani, 2011; Mohammadi et al., 2016; Takahashi et al., 2008; Parvan, Hosseini, & Bagherian, 2018).

With these problems, even if a nurse has a high degree of critical thinking, this thinking will be suppressed gradually, and he/she should be taken care of the patient in accordance with traditional nursing care. Also, they will not have an attitude towards the NP or that their attitude may be negative. Specifically, when the nurse does not have enough knowledge about the NP, there are no available facilities in the hospital, supervision and support for the NP is not available, and nurses will not have a good attitude towards the NP.

The use of the NP, especially the nursing diagnosis stage, allows nurses to use critical thinking for their clinical judgement and their clinical care activities (Ghanbari et al., 2017; Wolter Paans, Sermeus, Nieweg, & Van der Schans, 2010). The NP with critical thinking as a flexible tool and along with the contemporary integrate nursing philosophy ensures the high quality of care (Rahmani et al., 2016).

The existence of the NP by electronic means provides a valuable opportunity for nurses and nursing students to improve their clinical performance (Frigstad et al., 2015). Due to legal issues, nurses spend a lot of time writing their reports; if the NP is implemented electronically, this writing time will be reduced and nurses will have more time to evaluate the patient and pay attention to them (Semachew, 2018). Its implementation requires a lot of effort by nursing institutions and administrators because most nurses have little knowledge about the NP (Aein & Frouzandeh, 2012; Semachew, 2018).

The institutionalization of the NP in hospitals and its continuous implementation by nurses will depend on countries' economic, educational and access to care services. In the United States of America and Europe, this process despite numerous challenges runs manually or electronically (Di Mauro et al., 2018; Zamanzadeh et al., 2015).

The acceptance and emerge of this process in the clinical settings in the United States can be examined through two dimensions: sociological dimension and professional dimension (de la Cuesta, 1979). These two dimensions led to the massive movement to lead the nursing towards professional. The NP was a professional strategy for nurses in accordance with contemporary American society (de la Cuesta, 1983).

The acceptance and emergence of the NP in the high‐income countries including England, Italy, Switzerland, South Korea and China were related to the professional dimension. Dissatisfaction wave from the nursing care provided in these countries. Major factors of dissatisfaction include dissatisfaction of care provided by nurses, poor quality of care and nurses' dissatisfaction from their profession and denial of a task‐orientated approach to nursing. But with the implementation of the nursing process in these countries, many problems and dissatisfaction have solved (de la Cuesta, 1983; Semachew, 2018).

## 10 CONCLUSION

The NP as a scientific standard improves the quality and quantity of nursing care and documentation, and save time and cost with its implementation as electronically; improves nurse‐patient communication and with evidence‐based nursing care; and promotes critical thinking in nurses. Therefore, lower‐income countries must provide the necessary background for the implementation of this process.

## 11 RELEVANCE TO CLINICAL PRACTICE

Top managers and institutions of nursing should provide infrastructures such as e‐NP (electronically of NP) in the clinical settings; then, they will support the implementation of the NP, increase their supervision over the implementation of NP and increase nurses' motivation to nursing care based on the NP and designing a checklist for NP monitoring and attached them to patients' records. Therefore, appropriate policies must be adopted to implement the nursing process in the clinical settings of the lower‐income countries in the form of widely and continuously.

Considering the inadequate knowledge of nurses and faculty members about the NP, nursing diagnosis textbooks in accordance with the context of the country must be translated and used in clinical practice and nursing process training courses are also mandatory for graduate nurses.

An appropriate tool for assessing the quality of nursing care based on the NP is not psychometric, native software tailored to each country and in accordance with international standards in lower‐income countries has not been widely used, and the NP is still not implemented in these countries. Therefore, studies with instrumental research, software development and action research are necessary to institutionalize the NP.

## CONFLICTS OF INTEREST

No conflicts of interest declared.

## ETHICAL APPROVAL

Approval code of ethics with number: IR.TBZMED.REC.1397.170.
